# Comprehensive clone screening and evaluation of fed-batch strategies in a microbioreactor and lab scale stirred tank bioreactor system: application on *Pichia pastoris* producing *Rhizopus oryzae* lipase

**DOI:** 10.1186/1475-2859-13-36

**Published:** 2014-03-07

**Authors:** Johannes Hemmerich, Núria Adelantado, José Manuel Barrigón, Xavier Ponte, Astrid Hörmann, Pau Ferrer, Frank Kensy, Francisco Valero

**Affiliations:** 1 m2p-labs GmbH, Arnold-Sommerfeld-Ring 2, Baesweiler 52499, Germany; 2 Department of Chemical Engineering, Escola d’Enginyeria, Universitat Autònoma de Barcelona, Bellaterra, Barcelona 08193, Spain; 3 Centre for Genomic Regulation, Barcelona, Spain

**Keywords:** *Pichia pastoris*, Clone screening, *Rhizopus oryzae* lipase, Fed-batch fermentation, Feeding strategies, Microbioreactor, Scale-up, Bioprocess development

## Abstract

**Background:**

In *Pichia pastoris* bioprocess engineering, classic approaches for clone selection and bioprocess optimization at small/micro scale using the promoter of the alcohol oxidase 1 gene (P_*AOX1*_), induced by methanol, present low reproducibility leading to high time and resource consumption.

**Results:**

An automated microfermentation platform (RoboLector) was successfully tested to overcome the chronic problems of clone selection and optimization of fed-batch strategies. Different clones from Mut^+^*P. pastoris* phenotype strains expressing heterologous *Rhizopus oryzae* lipase (ROL), including a subset also overexpressing the transcription factor *HAC1*, were tested to select the most promising clones.

The RoboLector showed high performance for the selection and optimization of cultivation media with minimal cost and time. Syn6 medium was better than conventional YNB medium in terms of production of heterologous protein.

The RoboLector microbioreactor was also tested for different fed-batch strategies with three clones producing different lipase levels. Two mixed substrates fed-batch strategies were evaluated. The first strategy was the enzymatic release of glucose from a soluble glucose polymer by a glucosidase, and methanol addition every 24 hours. The second strategy used glycerol as co-substrate jointly with methanol at two different feeding rates. The implementation of these simple fed-batch strategies increased the levels of lipolytic activity 80-fold compared to classical batch strategies used in clone selection. Thus, these strategies minimize the risk of errors in the clone selection and increase the detection level of the desired product.

Finally, the performance of two fed-batch strategies was compared for lipase production between the RoboLector microbioreactor and 5 liter stirred tank bioreactor for three selected clones. In both scales, the same clone ranking was achieved.

**Conclusion:**

The RoboLector showed excellent performance in clone selection of *P. pastoris* Mut^+^ phenotype. The use of fed-batch strategies using mixed substrate feeds resulted in increased biomass and lipolytic activity. The automated processing of fed-batch strategies by the RoboLector considerably facilitates the operation of fermentation processes, while reducing error-prone clone selection by increasing product titers.

The scale-up from microbioreactor to lab scale stirred tank bioreactor showed an excellent correlation, validating the use of microbioreactor as a powerful tool for evaluating fed-batch operational strategies.

## Background

*Pichia pastoris* is recognized as an excellent expression system for heterologous protein production [1-3]. One of the main advantages of this cell factory is the use of a strong and tightly regulated promoter from the alcohol oxidase 1 gene, P_*AOX1*_[4]. This allows the use of methanol as sole carbon source as well as inducer for recombinant protein production. *P. pastoris* has two alcohol oxidase encoding genes (*AOX1* and *AOX2*), and three different phenotypes of *P. pastoris* host strains are available, according to their ability to metabolize methanol: the wild type (Mut^+^) and those resulting from deletions of *AOX1* gene, (Mut^s^) or both genes (Mut^–^) [5].

The standard procedure to achieve high cell densities and protein production is a fed-batch bioprocess using methanol as sole carbon source [6]. Nevertheless, the use of multicarbon substrate in addition to methanol is a common approach, especially for cultivations using Mut^s^ phenotype [7-9].

In the *P. pastoris* bioprocess based on P_*AOX1*_*,* clone selection is a critical bottleneck because reproducibility in shake flasks is rather low and, therefore, time consuming when the number of potential clones to screen is high [10]. The use of microtiter plates can increase the throughput of clone screening procedures, but reproducibility and scalability when using methanol is limited. Low reproducibility is mainly caused by “edge effects” [11], due to the uneven evaporation distribution throughout a microplate, especially in the outer wells of a microplate. This is observed in standard shakers without controlled atmosphere, e.g. for relative humidity. With the use of volatile substrates like methanol, this effect is even more pronounced. Furthermore, the optimization of fed-batch operational strategies for high cell densities can be expensive and time consuming. Although mathematical modelling can reduce the number of experiments, the application of new approaches to solve these drawbacks is necessary. In this context, the use of microbioreactors is an alternative to minimize these pitfalls.

Microbioreactors (MBR) are miniaturized versions of well-established bioreactor systems such as stirred tank fermenters (STR). Due to the micro-scale of these MBR, an exact scale-down of technical equipment is not possible in all cases. For example, tubing and pumps commonly used to feed nutrients or adjust pH are not commercially available or practical to handle the necessarily small volumes. Regarding MBR, other mechanisms have to be applied, like the integration of pipetting robots or microfluidic structures for liquid delivery to the fermentation broth [12-14].

Furthermore, mixing and aeration of fermentation broth in STR is usually achieved using mechanically agitated stirrers. In this respect, STR have been characterized for several decades [15-17]. In MBR, mixing and aeration is achieved by shaken microplates. Aeration is a critical parameter in cultivation of oxygen-demanding cell types like *E. coli* or yeast (*S. cerevisiae*, *P. pastoris*). In STR, oxygen transfer rate (OTR) is improved by increasing stirring and aeration rate, diminishing air bubble size and using pure oxygen or air enriched with oxygen instead of air. Similar strategies can be used partly for MBR, where an increase of the OTR has been achieved by means of new geometric design of the wells in shaken microtiter plates [18], or submerged injection of air/oxygen [19].

In this study, a RoboLector MBR system was used, which is the integration of the BioLector MBR system [20] into a liquid handling robot. This concept was described earlier [21]. The BioLector enables online monitoring of cultivation parameters from the incubated microplate (compare also Materials and Methods section). The online monitored signals, as well as run time and calculated actual volume, can serve as setpoints for the liquid handling unit to access the individual wells of the microplate. This enables the addition or removal of liquids once or periodically, which is used for sampling, induction or feeding of individual cultures.

Until now, only a few applications of MBR using *P. pastoris* in bioprocess development can be found in literature, although several laboratories have been using microtiter plates for clone screening purposes [22,23].

One MBR system was described for cultivation of *P. pastoris*[19]. For the feeding strategy used in that example, the cultivation cassette had to be removed from the machine and placed under a laminar flow cabinet to add several substrate shots manually. In contrast, the RoboLector platform is able to perform fed-batch operational strategies with conditions closer to those commonly applied in STR. Because of the integration of an automated liquid handling system in the RoboLector MBR, there is no need to remove the cultivation cassette (i.e. FlowerPlate) from the incubation machine (i.e. BioLector). Therefore, it is possible to add nutrients much more frequently without interruption of shaking and thus, without interruption of oxygen transfer. This high frequency of nutrient addition was a key parameter in mimicking a typical STR fermentation with *P. pastoris*. Additionally, the fed-batch strategy of enzymatic glucose release (with methanol induction every 24 h) is not restricted to the RoboLector MBR system. This strategy can be applied in any other MBR system like the BioLector stand-alone device.

In another study, Holmes et al. exploited the combinatorial use of a MBR system and design of experiments (DoE) methodology for optimizing specific yield in the induction phase of green fluorescent protein (GFP) [24]. They generated a predictive model for small-scale screens with the aim to prove the scalability to the bioreactors. This reduced development time and allowed focus on knowledge-driven optimization of feeding strategies. Process development was performed with a single clone compared to the study presented here with the RoboLector MBR system. Also, feeding strategies had to be optimized at the litter-scale in STR. In contrast, the RoboLector MBR system is able to perform fed-batch fermentations, allowing process development to shift into microscale.

Comparable to the approach of feeding nutrients at a high frequency, there is an example found in literature [25], where six parallel operated bubble columns with a working volume of several hundred milliliters were used. Due to the volume, this system should not be considered as a microbioreactor, but as a minibioreactor system. These bubble columns can be equipped with pO_2_- and pH-electrodes, while a pump delivers shots of 1 mL of methanol to the fermentation broth. Similar to the MBR RoboLector approach described here the authors report the usefulness of a scale-down approach to develop suitable process parameters, which are to be transferred into classical STR. It should be mentioned that this system required more resources for set up because of necessary cleaning and sterilization procedures, wiring and calibration of the electrodes and tubing set up for the pumping system.

Another kind of minibioreactor uses up to eight specialized shake flasks as culture vessels in parallel [26,27]. These flasks are equipped with caps having gas and pressure sensors, whereas the lower part is geometrically equal to standard shake flasks. The sensors determine respiration activities of the cultures, namely oxygen transfer rate (OTR), carbon dioxide transfer rate (CTR) and respiratory quotient (RQ). The authors recommend to culture replicates in standard shake flasks under same conditions, which serve for sampling and subsequent analytics. Also, there is technical equipment for sampling and feeding of the measuring flasks is available (HiTec Zang, Herzogenrath, Germany).

Data monitoring and manipulation of MBR cultivations are essential to generate process relevant results, especially when it comes to translating results into pilot and production scale. Finally, the aim of MBR studies in biotechnological developments is to shift as many steps as possible into the microliter scale. Therefore, suitable MBR systems have to operate in a reliable and robust way with user friendly handling to facilitate the high throughput needs.

With current techniques in molecular biology, huge clone pools are easily generated from combining the use of different genetic libraries (e.g. promoters [28,29], protein variants [30] or secretion signals [31]). In combination with different cultivation setups to be evaluated (e.g. feeding strategy, medium background, induction strength and optimal time point of induction), the resulting number of experiments grows very fast with each factor to be investigated [32]. Such extensive, combinatorial studies of clone screening and process optimization require methods of high-throughput. Additionally, the use of software tools like Design-of-Experiments (DoE) and genetic algorithms can boost performance, as the number of experiments to be conducted can be reduced in a meaningful way.

The aim of this study is to demonstrate that the RoboLector automated microbioreactor platform is a suitable tool to minimize the clone selection step and to optimize mixed substrates (methanol and other carbon source) fed-batch operational strategies for the P_*AOX1*_-based *P. pastoris* system. A set of Mut^+^ phenotype strains producing a heterologous *Rhizopus oryzae* lipase (ROL) was used as a case example. RoboLector was used as it matches some important requirements for bioprocess development [12,21].

## Results and discussion

### Clone selection

Two different series of X-33-derived strains expressing a lipase from *R. oryzae* (ROL) under the P_*AOX1*_ promoter were constructed. In the first series, a pre-existing X-33 strain expressing ROL [33] was transformed with an expression vector containing the induced form of the *P. pastoris’ HAC1* transcriptional factor under the control of P_*AOX1*_ (Clones 1–6). The second strain series was obtained by replica plating of X-33/pPICZαα_ROL transformants on agar plates containing increasing concentrations of zeocin, aiming at the selection of transformants with multiple copies of the *ROL* expression cassette (Clones 7–12). From each series of strains, six transformants were selected for further studies at MBR scale.

For reference and comparison purposes, expression experiments were initially performed in shake flasks, following a two-step procedure for Mut^+^ strains similar to the standard protocol described in the Invitrogen guidelines, that is, growing cells in minimal glycerol medium and subsequently transferring growing cells to a shake flask with fresh minimal methanol medium [34]. As previously described [35], ROL expression levels in shake flasks were rather low, close to the detection limit of the lipase activity assay, making it difficult to assess clonal variation and perform a reliable clone ranking.

### Microbioreactor cultivation

#### Effects of cultivation media in clone screening

**Figure 1:**
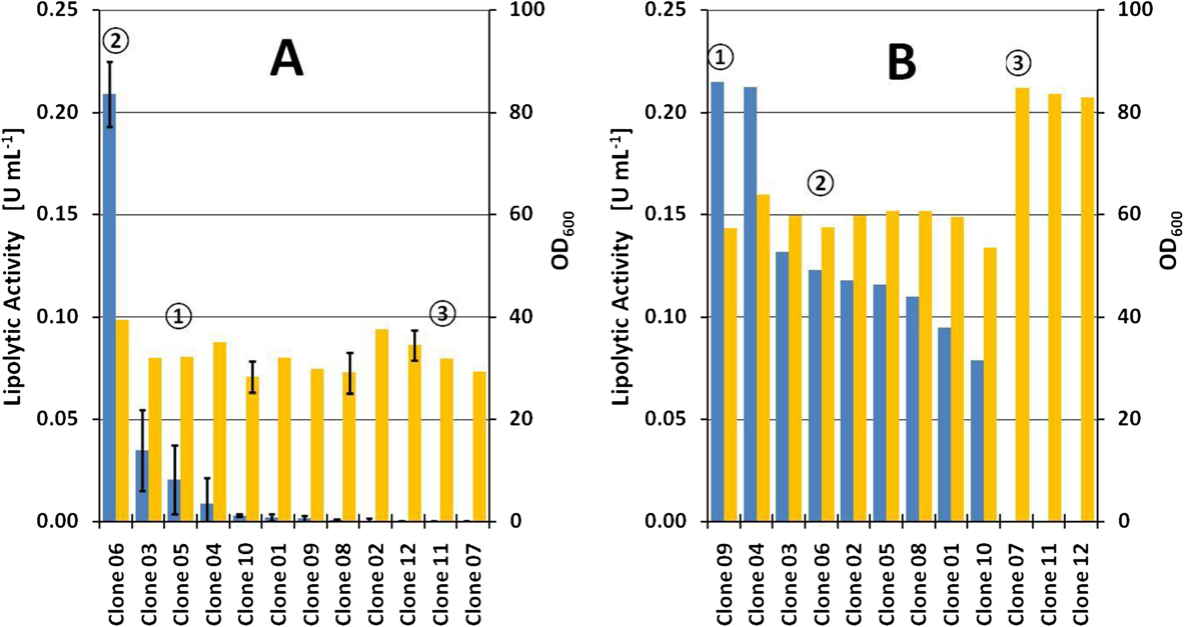
**Clone rankings obtained in YNB (A) after 80 h and Syn6 medium (B) after 72 h during MBR cultivations.** In blue, lipolytic activity and, in yellow, optical density at 600 nm. Numbers in circles indicate selected clones for further cultivation experiments. **A**: Mean values of three replicate wells. **B**: Values obtained from single well cultivations. Ranking criteria was volumetric lipolytic activity.

In order to check the effect of cultivation media in clone screening two different media were selected: YNB and Syn6. Clone screening was conducted similarly to the proposal in Invitrogen’s guide [34] and as applied often in literature [31,36-40]: Clones were grown in the selected media, following induction of P_*AOX1*_-driven expression by daily addition of methanol (0.5% v/v in YNB; 1.0% v/v in Syn6). Biomass concentrations are clearly higher in Syn6 medium, where the amount of both glycerol and methanol is higher compared to YNB medium (Figure 1). With YNB medium the biomass reached was similar in all the experiments. In contrast, for Syn6 medium clones with no detectable lipolytic activity exhibit clearly substantial higher biomass concentrations (≈ 80 OD_600_) compared to producing clones (≈ 60 OD_600_).

Syn6 medium also shows better performance in terms of lipolytic activity than YNB (Figure 1). Nine of twelve clones showed greater lipolytic activity than 0.05 U mL^-1^, but in YNB medium only one clone did. Interestingly, clone ranking based on lipolytic activity was different for both media backgrounds. Furthermore, clone ranking does not change if lipolytic activity is normalized to biomass concentration (U mL^-1^ OD_600_^-1^) or to methanol added (U mL^-1^ g^-1^_MeOH_).

In terms of clonal variation, the series of clones able to grow at higher zeocin concentrations, that is theoretically harbouring multiple ROL copies, showed greater variability than the *HAC1*-transformants series. Strikingly, some of the ROL multicopy clones (clones 11 and 12) produced almost no detectable activity in any of the growth conditions tested. Previous studies [35,41] have shown that ROL triggers the unfolded protein stress response (UPR), resulting in reduced biomass yields [42]. Moreover, recent studies suggest that increased *ROL* copy number could result in increased stress levels and, consequently, to a stronger reduction in biomass and product yields due to increase metabolic burden [43]. Therefore, undetectable lipolytic activity in clones 7, 11 and 12 was consistent with the observation that these clones reached higher biomass levels than the producing clones. This suggests that these clones might present some genetic modification(s) as a result of the transformation and clone selection process [44], which resulted in reduced or no active product formation.

In order to check the performance of the different clones in this screening, further bioprocess development with clones 4, 6 and 7 (as indicated in Figure 1) was conducted to justify the selection of a high-producing clone and to validate the scalability of the microbioreactor. Because Syn6 medium was used in further cultivation, clone ranking was adapted from results of batch screening in Syn6 medium. In contrast to YNB medium, Syn6 and its variants are known to promote growth to high cell densities [45].

#### Microbioreactor for evaluation of operational fed-batch strategies

With the possibility of in parallel, but independently operated cultivations in the microbioreactor system, two fed-batch strategies for the clones were evaluated at the same time. The first strategy was based on feeding glucose as main carbon source by the *in-situ* enzymatic release of glucose molecules from a soluble glucose polymer [46]. The glucose release rate is modulated by the amount of glucosidase added; also the glucose polymer cannot be metabolized by *P. pastoris*. To induce recombinant gene expression, methanol was added automatically to a final concentration of 1% v/v in intervals of 24 h.

The second strategy was implemented by the pulsed addition of a mixture of glycerol (main carbon source), methanol (inducer) and NH_4_OH (N-source) at two different feeding rates (see below).

In addition to biomass concentration and pO_2_, fluorescence of riboflavins and NAD(P)H were measured (Additional file 1). While riboflavin fluorescence increased with biomass concentration, NAD(P)H signal clearly responded to the addition of methanol. This indicates activity in the methanol assimilation pathway, which easily is revealed in the microbioreactor system due to the high frequency of fluorescence measurements (every 13 min). Also, fluorescence signal of NAD(P)H dropped shortly before pO_2_ rises, which indicates depletion of previously added methanol. Notably, these results are coherent with previous online and offline monitoring studies of *P. pastoris* fermentations [47]. This online information may be a useful tool to study methanol metabolism in further investigations, but is not scope of this study.

### Strategy 1: enzymatic continuous glucose feeding with MeOH induction

**Figure 2:**
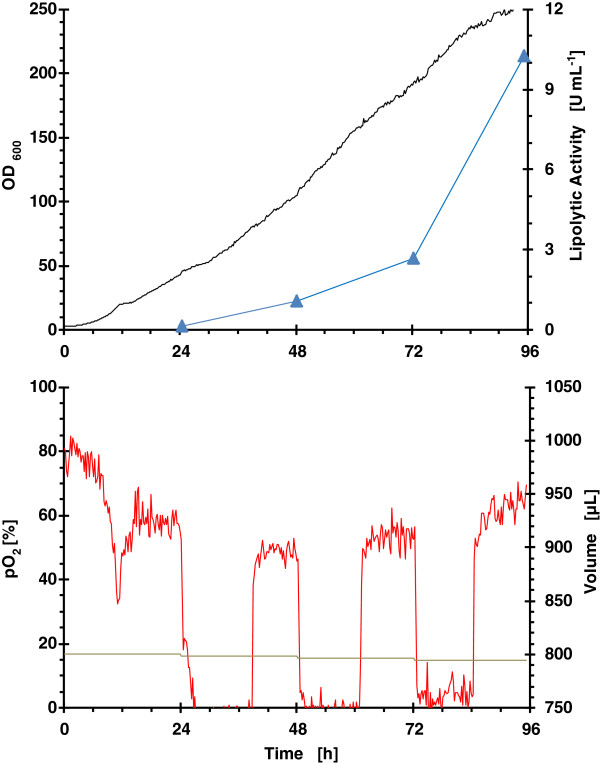
**Growth kinetics from RoboLector microbioreactor system for clone 4 (single well data).** Operational fed-batch strategy consisted on enzymatic feeding of glucose with MeOH addition in 24 h intervals. (black line) OD_600_; (blue filled triangle) lipolytic activity; (red line) pO_2_ and (grey line) volume.

The time course of microbioreactor cultures in terms of biomass, lipolytic activity, pO_2_ and accumulated volume is shown in Figure 2. Glucose is released at a nearly constant rate by enzymatic action on the glucose polymer. Although glucose concentration was not measured, the analysis of pO_2_ evolution indicates that at the beginning of the fermentation until approximately 12 h, where biomass concentration is low, glucose consumption rate is lower than glucose release rate and an accumulation of glucose should be produced. After that, pO_2_ levels are constant between methanol consumption phases and glucose-limited growth occurs, thus glucose concentration in the medium is close to zero.

The specific growth rate decreased along the fermentation from 0.035 to 0.012 h^-1^ as was expected due to the constant glucose release throughout the bioprocess. This low specific growth rate, far from the maximum value (0.2 h^-1^) helps the de-repression of P_*AOX1*_[42]. The specific growth rate can be controlled by modulating the quantity of glucose-liberating enzyme avoiding glucose accumulation. ROL is produced along the fermentation with the highest specific production rate during the last 24 hours.

The oxygen limitation observed after the addition of methanol diminished the specific methanol consumption rate of the microorganism, and also could affect to the specific production rate. However, an improvement of the production of monoclonal antibodies under oxygen-limited cultivation of glycoengineered yeast has been reported [48].

### Strategy 2: pulsed feeding of glycerol/MeOH

**Figure 3:**
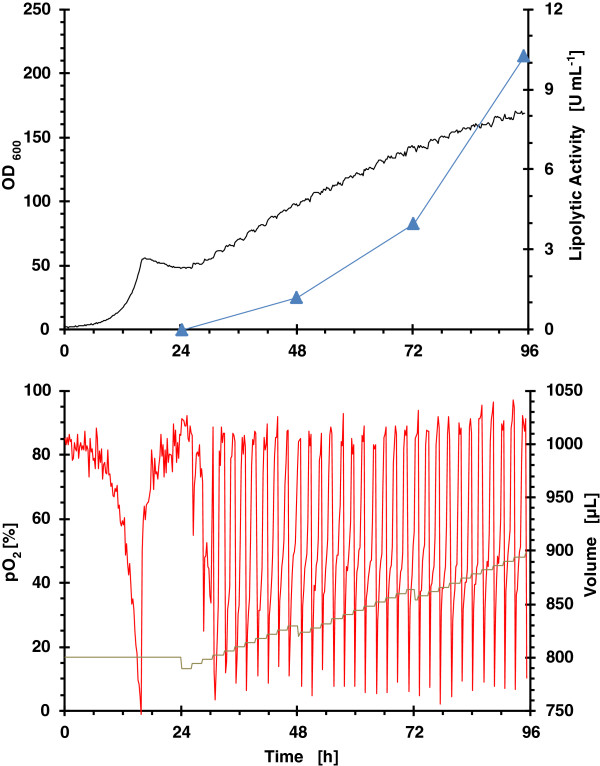
**Growth kinetics from RoboLector microbioreactor system for clone 4 (single well data).** Operational fed-batch strategy consisted on pulsed dosing of glycerol/MeOH at a rate of 2 μL h^-1^. (black line) OD_600_; (blue filled triangle) lipolytic activity; (red line) pO_2_ and (grey line) volume.

**Figure 4:**
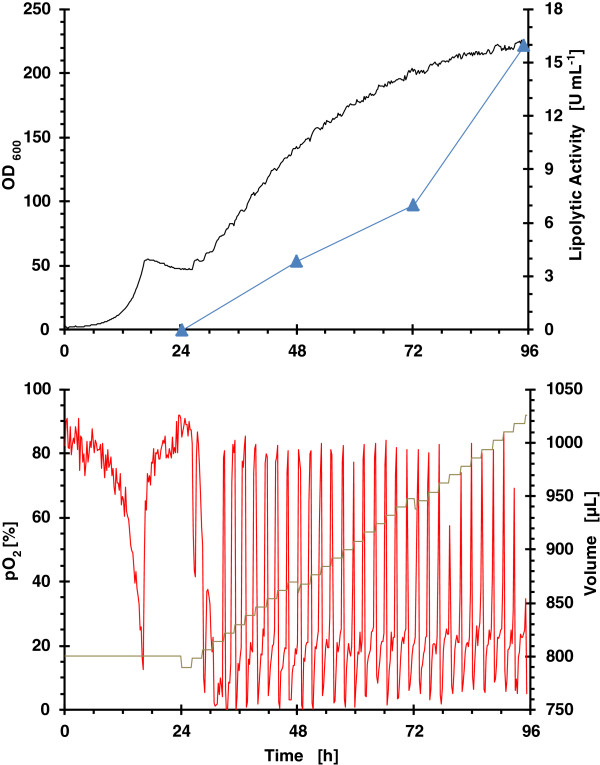
**Growth kinetics from RoboLector microbioreactor system for clone 4 (single well data).** Operational fed-batch strategy consisted on pulsed dosing of glycerol/MeOH at a rate of 4 μL h^-1^. (black line) OD_600_; (blue filled triangle) lipolytic activity; (red line) pO_2_ and (grey line) volume.

The performance of biomass, lipolytic activity, pO_2_ and accumulated volume from pulse addition of a mixed substrate (glycerol/MeOH) at low rate of 2 μL h^-1^, and high rate of 4 μL h^-1^, is presented in Figure 3 and Figure 4, respectively.

The pO_2_ time courses for both dosing rates demonstrate clearly, that the culture is able to metabolize previously added nutrients before the next substrate pulse bolus feed occurs. That means overfeeding does not occur.

As expected, the specific growth rate at low feeding rate is lower than at a high feeding rate which is also reflected in ROL activity over time. Again, as observed in cultivations with enzymatic feeding of glucose with MeOH induction, the highest increase in lipolytic activity occurs during the last 24 hours.

Although the different strategies applied have a notable influence on the production of heterologous product, the comparison in terms of biomass specific activities produced and activity yield from methanol is quite interesting (Table 1). In terms of biomass specific activity, high glycerol feeding rate is the best strategy: 1.2-fold higher than low glycerol feeding rate and 1.8-fold higher than glucose feeding. However, activity yield with respect methanol should be the key variable for the comparison between the three strategies, because methanol is the inducer for production and total methanol added was different for the three operational strategies. Comparing this parameter, glucose feeding is the best strategy, 1.3-fold higher than low glycerol feeding and 1.5-fold higher than high glycerol feeding. Thus, the methanol added is the key parameter in terms of maximizing ROL production. The importance of methanol added in ROL production was also confirmed using sorbitol as co-substrate [9]. Studies using glycerol as co-substrate in Mut^s^ phenotype producing ROL demonstrated that there is an optimal relationship of μ_Gly_/μ_MeOH_. When the relation is higher than the optimal, a decrease in specific activity is observed [49]. Under the glycerol feeding rates tested, the relationship of μ_Gly_/μ_MeOH_ was never higher than the optimal.

**Table 1:** Comparison of process variables, specific activities and yields for clone 4 under different fed-batch strategies in microbioreactor after 96 h

**Fed-batch strategy**	**Biomass [OD**_**600**_**]**	**Volumetric lipolytic activity [U mL**^**-1**^**]**	**Biomass specific activity [U mL**^**-1 **^**OD**_**600**_^**-1**^**]**	**MeOH added [mg]**	**Final volume [mL]**	**Activity yield from methanol [U mg**^**-1**^_**MeOH**_**]**
Glucose feeding	255	10.3	0.040	19	0.794	0.43
Low glycerol feeding	169	10.3	0.061	28.2	0.898	0.33
High glycerol feeding	222	16	0.072	56.4	1.026	0.29

#### Comparison of clone rankings for different fed-batch strategies in microbioreactor system

**Figure 5:**
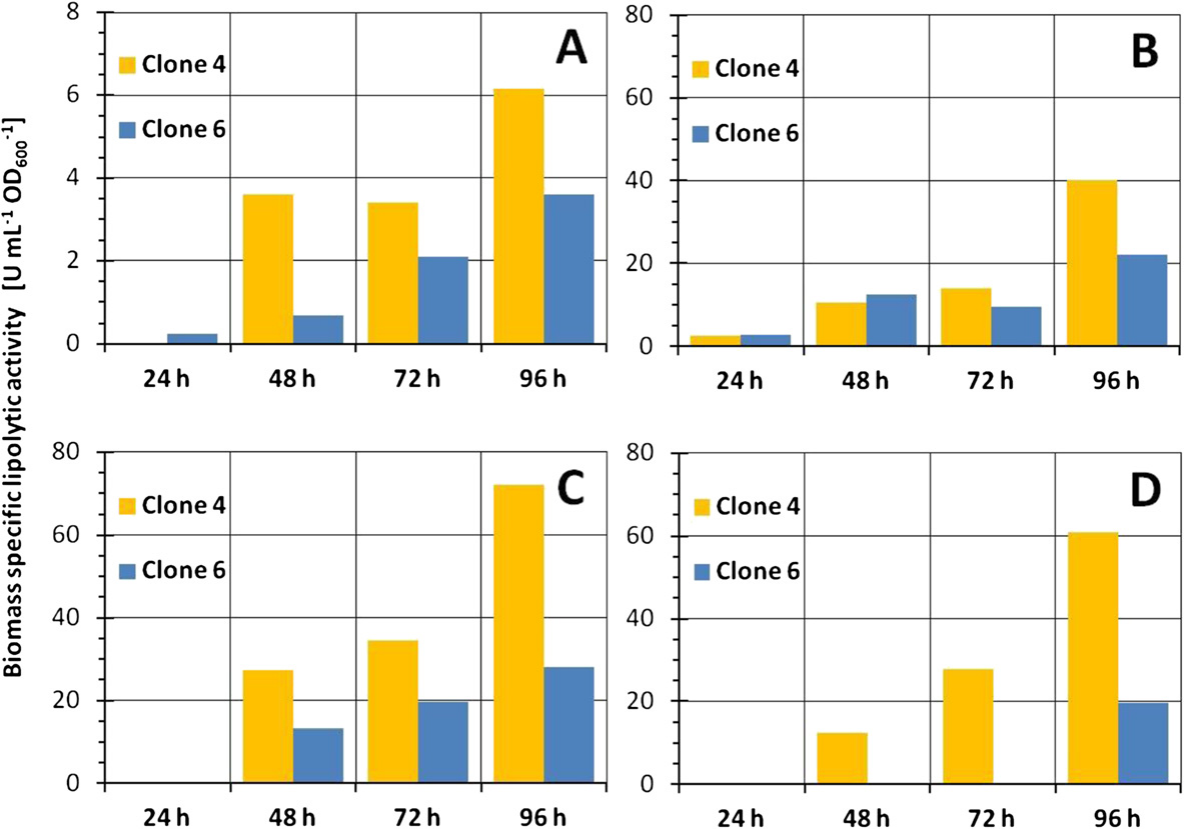
**Comparison of clone ranking for clones 4 and 6 obtained in different cultivation modes in RoboLector microbioreactor system (single runs).** Clone 7, which was also cultivated in STR next to clones 4 and 6, produced hardly any product and is therefore not shown in the figure. Medium background was Syn6 production medium. **A**: Batch screening with methanol induction. **B**: Enzymatic glucose feeding with methanol induction. **C**: Feeding of glycerol/methanol at 4 μL h^-1^. **D**: Feeding of glycerol/methanol at 2 μL h^-1^. Different scales are used in **A** (values between 0 to 8) and **B** to **D** (values between 0 to 80).

Finally, the performances of the three selected clones were compared for the three operational fed-batch strategies and batch bioprocess (Figure 5).

Interestingly, when the same background medium is used (Syn6 medium), the clone rankings are maintained between different cultivation modes, at least for the clones applied in this study. These results cannot be directly transferred to other hosts expressing other *genes-of-interest*, which highlights the need of tools like MBR when developing bioprocesses from scratch. This is supported by the observations in [50], where different clone rankings were obtained for two *H. polymorpha* clone libraries when cultivating in batch mode on glycerol, batch mode on glucose and fed-batch mode on glucose.

As expected, the lowest specific activities were observed in batch growth with subsequent MeOH addition every 24 hours, which were lower by one order of magnitude when compared with fed-batch strategies. The higher activities obtained in fed-batch cultivations facilitate the detection of the expressed product and at the same time ensures a more reliable clone selection.

### Lab scale bioreactor cultivations

**Figure 6:**
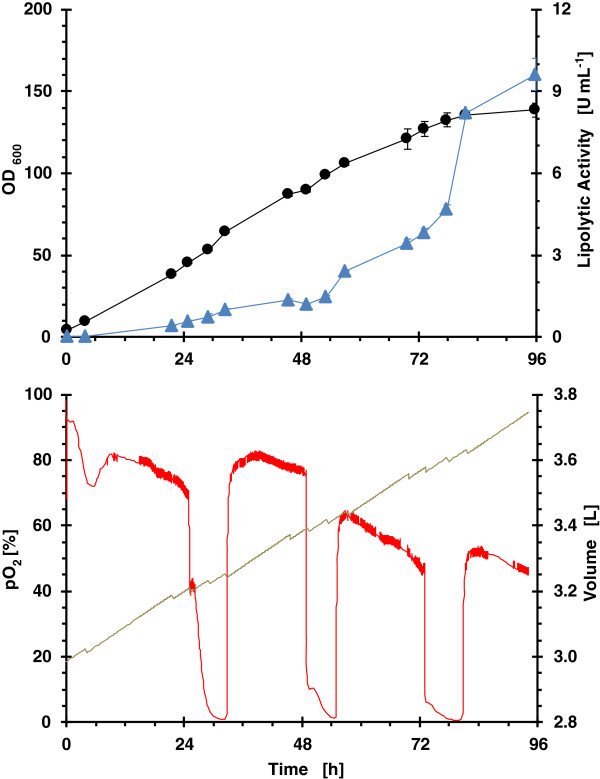
**Operational fed-batch strategy of constant feeding of glucose with MeOH addition in 24 h intervals in lab scale bioreactor cultivating clone 4.** (black filled circle) OD_600_; (blue filled triangle) lipolytic activity; (red line) pO_2_ and (grey line) volume.

**Figure 7:**
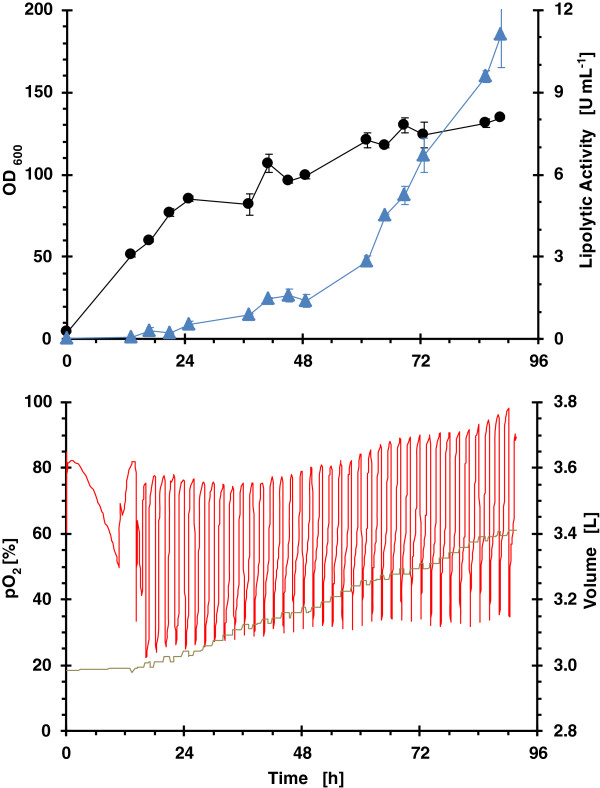
**Operational fed-batch strategy of pulsed addition of glycerol and MeOH in lab scale bioreactor cultivating clone 4.** (black filled circle) OD_600_; (blue filled triangle) lipolytic activity; (red line) pO_2_ and (grey line) volume.

In the present study, it is of interest to demonstrate if clone ranking and fed-batch operational strategies could be transferred from a microbioreactor unit (800 μL) to a classical stirred tank bioreactor (3 L), with a scale-up factor covering three orders of magnitude (factor >3000). Enzymatic glucose fed-batch mode and low feeding rate of glycerol/MeOH were the two selected strategies to compare scale up of the bioprocess with clone 4. The results obtained for both strategies are presented in Figure 6 and Figure 7.

#### Comparison of operational fed-batch strategies and scales

One of the targets of the bioprocess scale up was to obtain similar oxygen consumption profiles as to avoid differences in the ROL production could be attributed to the different oxygen transfer conditions. Aeration conditions in the lab bioreactor were chosen in order to get a similar OTR to that in the microbioreactor. OTR values between 50 mmol L^-1^ h^-1^ for a filling volume of 1100 μL and 65 mmol L^-1^ h^-1^ for a filling volume of 800 μL were determined with the method of sulphite oxidation [51] for the applied operating conditions, as specified in Materials & methods. Similar oxygen profiles were observed for enzymatic continuous glucose feeding in both bioreactors (Figure 2 and Figure 6). However, the oxygen profiles for pulsed feeding of glycerol are different (Figure 3 and Figure 7), in the bioreactor pO_2_ levels were always higher than 20%. However, in the microbioreactor, levels lower than 20% were observed.

The use of enzymatic release of glucose is an uncommon strategy for lab and industrial bioreactors, where feeding is realized by pumping concentrated nutrient solutions into the bioreactor. Enzymatic glucose release mimicks this approach without the need for additional technical equipment. In MBR the enzymatic glucose release was estimated around 1 g_Glucose_ L^-1^ h^-1^. This feeding rate was applied to the lab bioreactor with a constant 500 μL of glucose solution addition (300 g L^-1^) every 3 minutes. The final biomass concentration was lower in lab scale. According with this data, mean specific growth rate was 0.019 h^-1^ for STR *versus* 0.024 h^-1^ for MBR. Therefore, the glucose release rate of 1 g_Glucose_ L^-1^ h^-1^ for the MBR was a lower estimate than the real one.

Nevertheless, similar lipolytic activity values were reached at the end of the bioprocess for both bioreactors (Table 1 and Table 2). Although the total methanol added per cultivation volume was slightly lower in STR, activity yield with respect to methanol was slightly higher, 1.2-fold. This fact could be related to the lower specific growth rate reached in the lab bioreactor and subsequently a lower repression of P_*AOX1*_.

**Table 2:** Comparison of process variables, specific activities and yields for clone 4 under different fed-batch strategies in lab-scale bioreactor after 96 h

**Fed-batch strategy**	**Biomass [OD**_**600**_**]**	**Volumetric lipolytic activity [U mL**^**-1**^**]**	**Biomass specific activity [U mL**^**-1 **^**OD**_**600**_^**-1**^**]**	**MeOH added [mg]**	**Final volume [mL]**	**Activity yield from methanol [U mg**^**-1**^_**MeOH**_**]**
Glucose feeding	138 ± 4.2	9.8 ± 0.6	0.071	71010	3700	0.51
Low glycerol feeding	140 ± 0.1	12 ± 1.2	0.086	118350	3400	0.34

Whereas glycerol and sorbitol are frequent co-substrates used in heterologous protein production under P_*AOX1*_ promoter, the use of glucose as co-substrate is rarely described in literature due to the strong repression of the promoter [52]. Nevertheless, recent chemostat studies have shown the potential of glucose as a co-substrate for the P_*AOX1*_-based *P. pastoris* system under de-repressing conditions [42,53]. In contrast, the closely related methylotrophic yeast *Hansenula polymorpha* shows high expression rates when grown on glucose as sole carbon source, even if expression of the *gene-of-interest* is driven by a promoter originating from its MeOH-assimilation pathway [45]. However, at this low constant glucose feeding rate the amount of glucose available to the culture is taken up immediately. As shown in recent chemostat studies performed under carbon-limiting conditions [42,53], this “carbon starvation” may expose P_*AOX1*_ to de-repressing conditions, leading to full induction upon the addition of methanol.

The scale-up of low glycerol feeding rate fed-batch strategy, from an operational point of view was more successful due to the better reproducibility of the feeding profile. The time courses of OD_600_, lipolytic activity, pO_2_ and volume are presented in Figure 7. pO_2_ was maintained at values higher than 20%. However, in the microbioreactor (Figure 3), values lower than 20% were reached at the beginning of every mixed substrate addition pulse. Although it should be reflected in the profile of lipolytic activity, no significant differences were observed in terms of lipolytic activity and activity yield with respect to methanol (5 - 15% difference).

In terms of biomass specific activity, this value was 1.2-fold higher in the lab bioreactor than in the microbioreactor. A plausible explanation for such differences would be caused by the different dissolved oxygen profiles, since oxygen availability affects methanol assimilation rate and, in particular, *AOX1* transcriptional levels [54], even in glucose-only growth conditions [55]. Transient oxygen-limiting conditions observed in the microbioreactor cultivations after each methanol pulse may result in a reduction of *AOX1* transcriptional levels, as previously shown in shake flask cultures equipped with pO_2_ online monitoring [56].

**Figure 8:**
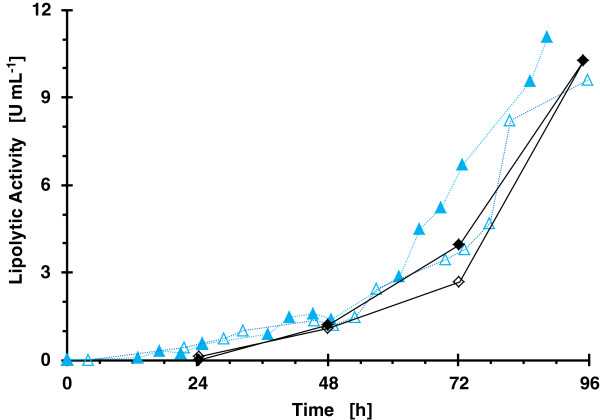
**Comparison of fed-batch operational strategies and scales in respect to volumetric lipolytic activity for cultivations with clone 4 (X33-ROL-Hac1_sc5).** (blue filled triangle) refers to lab scale bioreactor with glycerol/MeOH feeding, (blue empty triangle) to lab scale bioreactor with glucose and MeOH feeding, (black filled diamond) to microbioreactor with glycerol/MeOH feeding and (black empty diamond) to microbioreactor with glucose and MeOH feeding.

The comparison of the lipolytic activity values reached for both strategies and bioreactors is presented in Figure 8. The patterns of lipolytic activity time courses are quite similar, with a difference of less than 10% for the final lipolytic activity.

**Figure 9:**
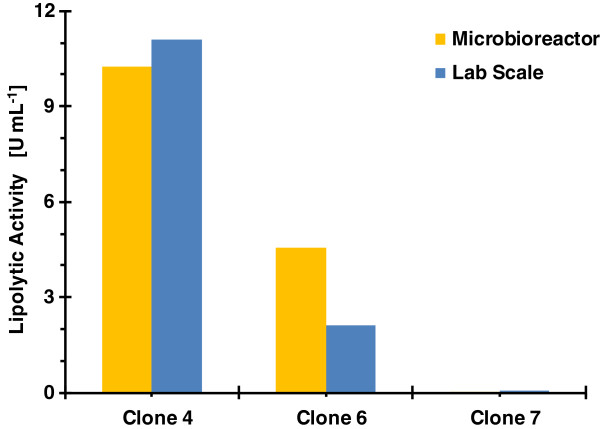
**Comparison of clone ranking after 96 h for microbioreactor and lab scale.** Operational fed-batch strategy was feeding of glycerol/MeOH at low rate. Ranking criteria was final volumetric lipolytic activity.

For the verification of clone ranking in lab scale, clones 4, 6 and 7 were tested at low glycerol feeding rate fed-batch strategy. The lipolytic activity at 96 hours for both scales is shown in Figure 9. Not only the ranking was maintained but also the activity levels reached were comparable. These results are quite remarkable because in many cases clone selection made by conventional approaches like shake flask cultivations do not correspond with the production expectations when they are tested at lab or pilot plant scale.

## Conclusions & outlook

The results presented in this study demonstrate the feasibility of the RoboLector MBR system in bioprocess development with *P. pastoris* as microbial cell factory. In particular, the implementation of fed-batch strategies in microbioreactors has demonstrated the reliable performance for clone selection with the P_*AOX1*_-based *P. pastoris* system using methanol as inducing substrate. Also, the results prove that the RoboLector platform is compatible with the use of a volatile and high O_2_-demanding substrate such as methanol. Furthermore, different operational fed-batch strategies at microscale for *P. pastoris* and clone screenings were performed and evaluated. Results were scalable to the conventional lab scale stirred tank bioreactor, covering three orders of magnitude (factor >3000). In addition, the influence of media composition in clone selection was demonstrated. The capabilities of the RoboLector MBR system accounted for the success of the study: online monitoring of relevant fermentation parameters, integration of pipetting robot for manipulation of cultures at a high frequency based on online monitored data, utilization of FlowerPlates allowing up to 48-fold parallel cell culturing at elevated oxygen transfer rates and scalability of clone ranking and fed-batch operational strategies (particularly mixed feeds) into classical STR. The development of these strategies may also be suitable for clone screening and fermentation development with other industrially important expression hosts.

In conclusion, the exciting area of MBR systems is still in motion to further expand the possibilities of such systems. Currently, there are more and more analytical systems which are designed to perform tasks for bioprocess development in a high throughput manner. In future, the integration of such machines into already existing sophisticated systems like the RoboLector makes MBR even more powerful and will contribute to next-level biotechnological developments until complete upstream and downstream processing can be executed by MBR systems.

## Methods

### Organisms

The *P. pastoris* X-33/pPICZαROL strain [33] was used as starting strain to generate a series of transformants co-overexpressing the spliced form of *HAC1* from *P. pastoris*. Isolation of the spliced form of *HAC1* from *P. pastoris* was performed following a strategy based on [57]. Briefly, the intronless *HAC1* cDNA was isolated from an exponential phase *P. pastoris* GS115 (Invitrogen) culture incubated in the presence of 10 mM DTT for 3 h to induce the unfolded protein response (UPR). Total RNA from the UPR-induced cultures was isolated using the Qiagen RNeasy Mini kit following manufacturer’s instructions. Reverse transcriptase-PCR was performed with the Titan 1 Tube RT-PCR System (Roche) using the forward primer 5’-ATGCCCGTAGATTCTTCTCATAAGACAGC-3’ and the reverse primer 5’-CTATTCCTGGAAGAATACAAAGTC-3’. The resulting PCR fragment was purified and cloned into pJET1.2/blunt (Fermentas CloneJet PCR Cloning kit) according to the sticky-end cloning protocol, using *E. coli* DH5α as host strain. In order to introduce a *Xho*I site and Kozac sequence and, a *Not*I site at the 5’ and 3’ ends, respectively, of the cloned PCR fragment, a second PCR was perfomed using pJET-HACspliced as template and employing the forward primer 5’-CATGA***CTCGAG***ACCATGCCCGTAGATTCTTCTCATAAGAC-3’ (*Xho*I site underlined) and the reverse primer 5’-TTAAA***GCGGCCGC***CTATTCCTGGAAGAATACAAAGTCATTTAAATC-3’ (*Not*I site underlined). The resulting PCR fragment was digested with *Xho*I and *Not*I and cloned behind the AOX1 promoter in the *Xho*I/*Not*I linearized pPIC3.5 K plasmid (Invitrogen). The resulting plasmid was named pPIC3.5 K-*HAC1spliced*.

Competent *P. pastoris* cells were prepared and transformed according to [58]. The pPIC3.5 K-*HAC1spliced* was linearized in the *HIS4* gene with *Nco*I for integration targeting of the construct in this locus. Transformants were plated on YPD agar plates containing 250 mg L^-1^ geneticin. In order to verify the integration of the *HAC1spliced* cassette, a PCR was performed on purified genomic DNA of the transformants with the forward primer 5’-GACTGGTTCCAATTGACAAGC-3’ (*AOX1* promoter region) and the reverse primer 5’-GCCGCCTATTCCTGGAAGAATAC-3’, with the following cycling conditions: 2 min 95°C, followed by 35 cycles of 45 s at 94°C, 45 s at 55°C, 1 min 72°C.

The series of strains having multiple copies of the *ROL* expression cassette was obtained by transforming X-33 (Invitrogen) competent cells with pPICZαA-ROL [33], as described in [42]. ROL gene dosage of these clones was not further determined.

### Cultivation media

Syn6 production medium contained per liter: 20 g glycerol; 7.66 g (NH_4_)_2_SO_4_; 9 g K_2_HPO_4_; 3.3 g KCl; 3 g MgSO_4_ · 7 H_2_O; 0.33 g NaCl; 0.1 mol MES; 4.202 g citric acid · H_2_O; 1 g CaCl_2_ · H_2_O; 10 mL vitamin solution; 10 mL micro elements solution and 10 mL trace elements solution. Vitamin solution contained per 100 mL: 20 mg d-Biotin and 2 g Thiamine · HCl. Micro elements solution contained per 100 mL: 1 g (NH_4_)_2_Fe(SO_4_)_2_ · 6 H_2_O; 0.08 g CuSO_4_ · 5 H_2_O; 0.3 g ZnSO_4_ · 7 H_2_O; 0.4 g MnSO_4_ · H_2_O and 1 g Titriplex III. Trace elements solution contained per 20 mL: 2 mg NiSO_4_ · 6 H_2_O; 2 mg CoCl_2_ · 6 H_2_O; 2 mg H_3_BO_3_; 2 mg KI and 2 mg Na_2_MoO_4_ · 2 H_2_O. The pH-value of the medium was adjusted to 6.4 with KOH. Cultivations with enzymatic glucose release were conducted with a proprietary formulation based on a Syn6 medium containing a soluble glucose polymer which cannot be metabolized by *P. pastoris*. In-situ glucose release is realized by addition of a glucosidase, which breaks the glucose polymer into single glucose units. Release rate of glucose can be adjusted with amount of added glucosidase (M-KIT-100, m2p-labs, Baesweiler, Germany).

YNB screening medium contained per liter: 10 g glycerol; 1.34 g Yeast Nitrogen Base without amino acids and ammonium (Difco); 5 g (NH_4_)_2_SO_4_; 0.4 mg d-Biotin and 0.1 mol Na_2_HPO_4_/NaH_2_PO_4_ (pH 6.0).

YPD preculturing medium contained per liter: 10 g yeast extract (Merck), 20 g peptone (Merck), 20 g glucose.

### Shake flasks cultivations

Triplicate shake flask cultures (250 mL nominal volume) were performed as follows: 25 mL of buffered minimal glycerol (BMG) medium were inoculated with a fresh colony and incubated over night at 25°C and 150 rpm (Infors Shaker, 25 mm shaking diameter). After 20 hours, cells were centrifuged (3000 × g, 5 min) and resuspended into 25 mL buffered minimal methanol (BMM) medium to an initial OD_600_ of 1.0. After 24 hours of induced expression, OD_600_ and lipolytic activity of the cultures were measured.

### Microbioreactor cultivations

The microbioreactor was a RoboLector system, which consists of a BioLector device (G-BL-100, m2p-labs, Baesweiler, Germany) integrated into a Multiprobe II Ex liquid handling robot (PerkinElmer, Waltham MA, USA). The BioLector device monitored from a incubated microplate the following parameters for each well of the microplates within a measurement interval of 13 min: scattered light (proportional to biomass concentration), Riboflavin fluorescence (λ_Ex._ = 488 nm, λ_Em._ = 520 nm), NAD(P)H fluorescence (λ_Ex._ = 365 nm, λ_Em._ = 450 nm) and dissolved oxygen tension (pO_2_) via integrated optodes in the bottom of the microplate’s wells. The incubation chamber of the BioLector device controlled relative humidity above 85% to minimize evaporation from the microplate’s wells. Scattered light readings were calibrated to OD_600_ as follows: At the end of the RoboLector runs, biomass values were obtained by measuring optical density at 600 nm of fermentation broths in all wells of the FlowerPlates, resulting in a linear relationship between scattered light and OD_600_.

Cultivations were carried out exclusively in 48 well FlowerPlates (MTP-48-BO, m2p-labs, Baesweiler, Germany), shaking frequency of 1100 rpm, shaking diameter of 3 mm, initial volume of 800 μL per well, maximum allowed volume of 1100 μL per well due to volume increase caused by feeding. Cultivation temperature was 28°C. FlowerPlates were sealed with a gas permeable membrane with a pre-slitted silicone layer (F-GPRS48-10, m2p-labs, Baesweiler, Germany) for penetration by robotic tips. MeOH addition of 8 μL was programmed in 24 h intervals for cultivations conducted in batch mode and enzymatic glucose fed-batch mode. For batch cultivation in YNB-Medium, methanol was added to a final concentration of 0.5% v/v. Feeding of the nutrient mixture (200 g L^-1^ glycerol, 25% v/v MeOH and 1.5% w/w NH_4_OH) was programmed to start after 24 h with a pulsing rate of 4 μL or 8 μL every 2 h (i.e. 2 μL h^-1^ or 4 μL h^-1^). Automated sampling was programmed in 24 h intervals for all cultivation modes. Feeding and sampling was performed without interruption of shaking and thus, avoiding interruption of oxygen transfer and sample deviations caused by settling cells. Sampling volume was 10 μL. Assay of lipolytic activity was performed immediately at-line to MBR cultivations after sampling. Cultivations were started with initial OD_600_ of 2.5, inoculated from pre-cultures grown overnight in 20 mL YPD medium in 250 mL Erlenmeyer flasks, at a shaking frequency of 250 rpm, a shaking diameter of 25 mm and 28°C.

### Bioreactor cultivations

Pre-cultures for bioreactor cultures were grown for 24 h in 1 L baffled shake flasks at 30°C, 150 rpm, in YPD medium containing 1 mL of a zeocin solution (100 mg mL^-1^, InvivoGen). Shake flasks contained 200 mL of YPD medium. The culture was centrifuged at 8000 rpm, 15 min and the harvested cells were re-suspended in bioreactor culture medium and used to inoculate a 5 L Biostat B bioreactor (Braun Biotech, Melsungen, Germany) at an initial optical density of 3.

Cells were cultured under the following cultivation conditions: initial volume 3 L, stirring rate 600 rpm, temperature 28°C, pH controlled at 5.0 by adding NH_4_OH 30% (v/v), air flow rate 3 L min^-1^. The cultivation started with a 20 g L^-1^ glycerol batch phase. When glycerol was exhausted, fed-batch phase was initiated, lasting for approximately 72 hours.

### Biomass analysis

Biomass analysis was performed by measuring triplicates of the optical density at a wave length of 600 nm in cuvettes of 1 cm path length.

### Lipolytic activity assay

Samples from MBR cultivations were diluted with phosphate buffered saline (PBS) and analyzed for lipolytic activity at 30°C in 96 well microplates using a TECAN microplate reader pre-heated to 30°C. Dilution factor of samples was 20 and resulted in a linear increase of absorption at 410 nm for at least five minutes. 10 μL of diluted sample were mixed with 190 μL of freshly prepared reaction mix (1 volume of 30 mg p-nitrophenylpalmitate (pNPP) in 10 mL isopropanol and 9 volumes of 90 mL potassium phosphate buffer (100 mM, pH 8) containing 111.1 mg gum arabic and 207 mg sodium deoxycholate). For calculation of released amount of p-nitrophenol (pNP) from pNPP under assay conditions, a calibration was done by using 10 μL of a pNP solution with known concentrations. Measurement interval of absorption readings at 410 nm was set to 45 s, with 10 s of shaking before each measurement. Increase in absorption due to autohydrolysis of pNPP could not be detected during measurement time. Volumetric activity under assay conditions for the release of 1 μM of pNP per min per mL of sample volume was calculated as follows: Activity [U mL^-1^] = ΔA_410 nm_ [a.u. min^-1^] * Slope of pNP-calibration [μmol_pNP_ L^-1^ a.u.^-1^] * dilution factor * 0.001.

For bioreactor cultures, extracellular lipolytic activity was measured by using a p-nitrophenylbutyrate (pNPB) assay. Cells were removed by centrifugation (13,000 rpm, 3 min). Then, samples were diluted with PBS and lipolytic activity was followed spectophotometrically in a cary Varian 300 spectophotometer (Varian Inc., Palo Alto, USA) at 30°C after mixing in a 1 mL cuvette 40 μl of sample and 960 μL of freshly prepared, pre-warmed reaction mix (1 volume of 19 mg pNPB in 10 mL isopropanol mixed with 9 volumes of 250 mM Tris-HCl, pH 7.5). Linear increase of absorption at 410 nm was followed for five minutes. Volumetric activity under assay conditions for the release of 1 μM of pNP per min per mL of sample volume was calculated as follows: Activity [U mL^-1^] = ΔA_410 nm_ [a.u. min^-1^] * Slope of pNP-calibration [μmol_pNP_ L^-1^ a.u.^-1^] * dilution factor * 0.001. In order to compare data a correlation between both methods (pNPP and pNPB) was conducted, applying a ROL dilution series with known concentration in the two methods.

### Determination of oxygen transfer rates in microbioreactor system

Oxygen transfer rates were determined by sulphite oxidation according to the method described by Hermann et al. [51]. After completion of the oxidation reaction, a down-shift in pH occurs, which is visualized by a pH indicator and thus, the end of oxidation reaction can be determined by a color change from blue to yellow. In contrast, when OTR determination based on this method is performed in FlowerPlates with optodes for pO_2_ sensing, end of oxidation reaction can be detected directly by an increase in pO_2_ signal (i.e. when there is no sulphite left to be oxidized). Thus, pH indicator was omitted. OTR determination was conducted at least in triplicates for each filling volume at a temperature of 25°C.

## Abbreviations

a.u.: Arbitrary units; MBR: Microbioreactor; MeOH: Methanol; ROL: *Rhizopus oryzae* lipase; OTR: Oxygen transfer rate; STR: Stirred tank reactor.

## Competing interests

The authors declare that they have no competing interests.

## Authors’ contributions

JH: RoboLector microbioreactor cultivations and corresponding setups and analytics. Interpretation and discussion of results from microbioreactor cultivations. Writing the manuscript. FK: Supervision of microbioreactor cultivations and overall conceptual design of the study, interpretation and discussion of the results and drafting of the manuscript. AH: Isolation of the HAC1 spliced form from *P. pastoris* and cloning into pPIC3.5 K. JMB, NA and XP: Bioreactor Fermentations and result discussion. PF, FV: Overall conceptual design of the study, interpretation and discussion of the results and drafting of the manuscript. All authors read and approved the final manuscript.

## Supplementary Material

Additional file 1 **Comprehensive graphs of microbioreactor and stirred tank bioreactor cultivations for clone 4 will all monitored data (Biomass, lipolytic activity, pO**_**2**_**, volume, NAD(P)H, riboflavin).** Click here for file
