# Supplementary material for: Comprehensive clone screening and evaluation of fed-batch strategies in a microbioreactor and lab scale stirred tank bioreactor system: application on Pichia pastoris producing Rhizopus oryzae lipase
Source: Microb Cell Fact. 2014 Mar 7;13:36. doi: 10.1186/1475-2859-13-36 (PMC4007594; doi:10.1186/1475-2859-13-36)
Supplement: Additional file 1 — Comprehensive graphs of microbioreactor and stirred tank bioreactor cultivations for clone 4 will all monitored data (Biomass, lipolytic activity, pO 2 , volume, NAD(P)H, riboflavin). [file 1475-2859-13-36-S1.docx]

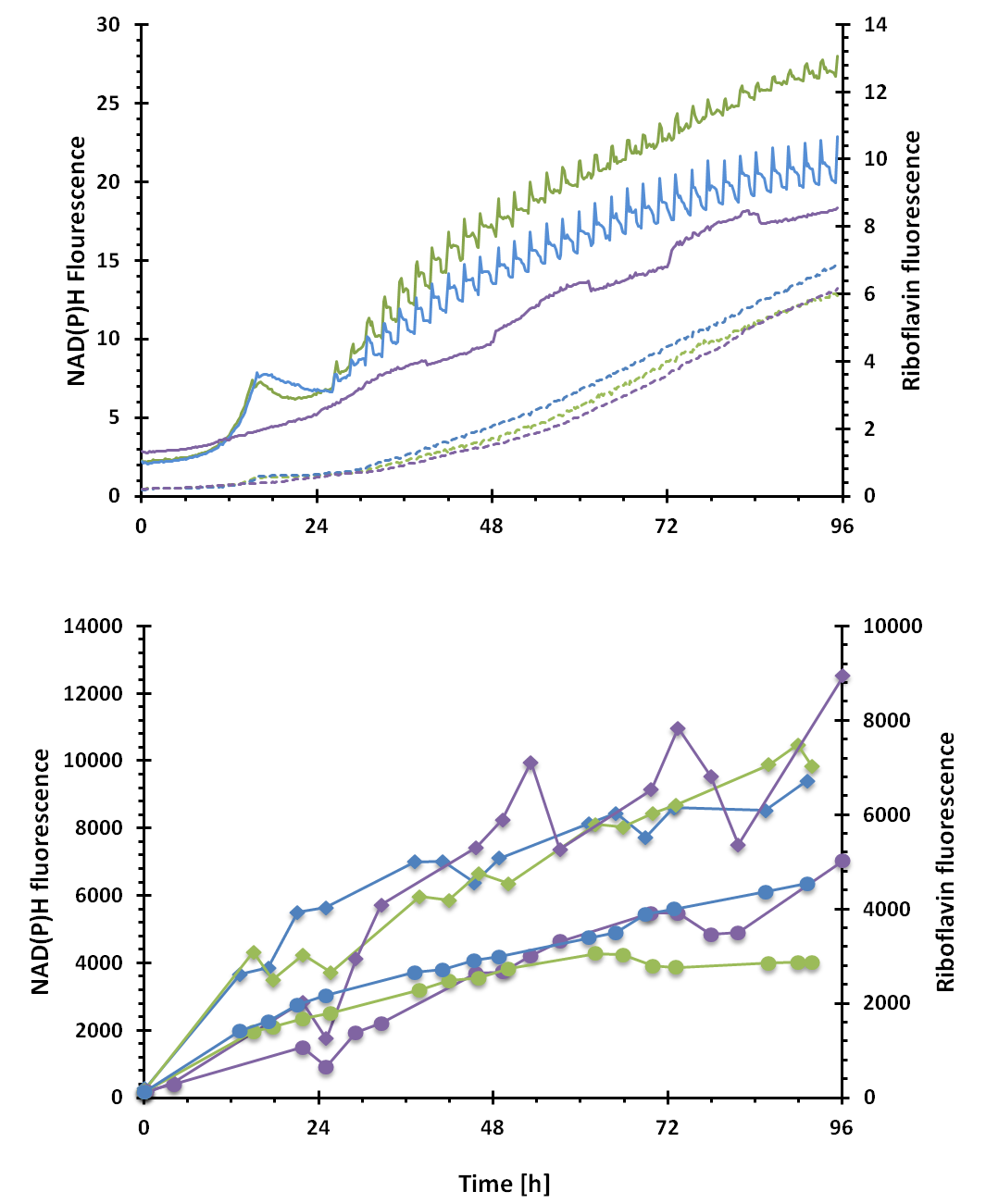


**Additional file 1: Figure S1:** Above, online NAD(P)H (
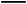
) and riboflavin (
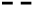
 ) fluorescence obtained during the performance of MBR cultures. Below, offline NAD(P)H (
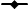
) and riboflavin (
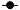
) fluorescence obtained in STR cultures. For both representations, blue refers to the strategy of glycerol/MeOH addition using clone 4, green to the one of glycerol/MeOH addition with clone 6 and, finally, purple refers to the strategy of glucose feeding with clone 4.
